# Pillow Support Model with Partitioned Matching Based on Body Pressure Distribution Matrix

**DOI:** 10.3390/healthcare9050571

**Published:** 2021-05-12

**Authors:** Yu Li, Jianfeng Wu, Chunfu Lu, Zhichuan Tang, Chengmin Li

**Affiliations:** 1 Industrial Design and Research Institute, Zhejiang University of Technology, Hangzhou 310023, China; silent.lee@126.com (Y.L.); luchunfu@126.com (C.L.); 2 College of Design and Architecture, Zhejiang University of Technology, Hangzhou 310023, China; ztang@zjut.edu.cn (Z.T.); chengmin517@126.com (C.L.)

**Keywords:** recumbent ergonomics, body pressure distribution, comfort model, ergonomics, product design

## Abstract

(1) Objective: Sleep problems have become one of the current serious public health issues. The purpose of this research was to construct an ideal pressure distribution model for head and neck support through research on the partitioned support surface of a pillow in order to guide the development of ergonomic pillows. (2) Methods: Seven typical memory foam pillows were selected as samples, and six subjects were recruited to carry out a body pressure distribution experiment. The average value of the first 10% of the samples in the comfort evaluation was calculated to obtain the relative ideal body pressure distribution matrix. Fuzzy clustering was performed on the ideal matrix to obtain the support surface partition. The ideal body pressure index of each partition was calculated, and a hierarchical analysis of each partition was then performed to determine the pressure sensitivity weight of each partition. Using these approaches, the key ergonomic node coordinates of the partitions of four different groups of people were extracted. The ergonomic node coordinates and the physical characteristics of the material were used to design a pillow prototype. Five subjects were recruited for each of the four groups to repeat the body pressure distribution experiment to evaluate the pillow prototype. (3) Results: An ideal support model with seven partitions, including three partitions in the supine position and four partitions in the lateral position, was constructed. The ideal body pressure distribution matrix and ideal body pressure indicators and pressure sensitivity weights for each partition were provided. The pillow that was designed and manufactured based on this model reproduced the ideal pressure distribution matrix evaluated by various groups of people. (4) Conclusion: The seven-partition ideal support model can effectively describe the head and neck support requirements of supine and lateral positions, which can provide strong support for the development of related products.

## 1. Introduction

According to a survey conducted by the World Health Organization, the incidence of insomnia among Chinese nationals is as high as 38%. Sleep problems have become a serious public health issue [1] as they can cause serious negative impacts on mental and physical health [2]. A pillow can provide reasonable support for the head and neck and help people maintain good neck and thoracic curvature in sleeping positions. Studies have shown that a comfortable sleeping pillow can relax the neck muscles to help people fall asleep and also effectively reduce pain in the neck, shoulders, back, and head [3].

Current research on the comfort of supporting surfaces mainly focuses on the sitting posture, and relatively few studies have examined the lying posture. The ergonomics of the head and neck while sleeping are relatively complex compared to the ergonomics of the body while sitting. The differences lie mainly in the following three aspects. First, the human body can assume many different positions while lying down, and the curvature of the contact support surface changes greatly among these positions, so the support requirements for the head and neck in these positions are significantly different [4]. Second, the supporting surface is in contact with the human head and neck area, and the subcutaneous physiological structure is complex, so different regions of the area have different sensitivity to pressure. Third, due to the relative relaxation of muscles and brain during sleep, electromyogram (EMG) and electroencephalogram (EEG) are not strong indications of comfort. Therefore, the comfort evaluation standard is unclear.

Current research on pillows focuses roughly on two areas: comparative study of subjective and objective evaluation of static comfort, and comfort evaluation prediction based on computer algorithms.

The evaluation methods of static comfort are divided into subjective evaluation and objective evaluation. Subjective evaluation refers to the evaluation of comfort by filling out a subjective evaluation form after a sleep test. Although this method is direct, subjective factors can easily interfere with the results. The repeatability is poor, the experiment is complicated, and it takes a long time to complete the evaluation [5]. Objective evaluation refers to analyzing the comfort of the supporting surface through data recorded by instruments, such as EMG signals [6,7], body pressure distribution [8,9], electrocardiogram, and anthropometry [10]. The combination of subjective and objective evaluation can effectively evaluate the comfort level of the supporting surface. Studies have found that among a large number of objective evaluation methods, the body pressure distribution has the most significant characterization effect on the comfort of the support surface of the human body [11]. Comfort factors, such as support material, support shape, support layout parameters, and human weight, can all be reflected in the body pressure distribution. Body pressure distribution is widely used in the objective evaluation of the comfort of various ergonomic support surfaces, including pillows, which are combined with subjective evaluation to study the comfort of ergonomic support surfaces.

In terms of the comfort prediction of support surfaces, comfort prediction models based on algorithms, such as stepwise multiple linear regression [12], back-propagation (BP) neural network [13,14], and support vector machine [15], are more commonly used. However, the prediction models obtained by the above methods still need to be improved in terms of accuracy and operating efficiency. For example, the linear regression method can hardly reflect the relationship between periodicity and nonlinearity. The support vector machine algorithm lacks methods for determining the kernel function. Although the BP neural network is widely used in predicting comfort, it does have limitations, such as high sensitivity to initial weights, likelihood of falling into a local optimum during optimization, and overfitting.

In addition, about 20–40% of the human body skin is in a state of stress when sleeping. Studies have shown that long-term improper pressure on specific areas of the human body can affect the human central nervous system [16,17,18], blood circulatory system [19,20], and endocrine system [21,22]. In addition, different areas of the human body show great differences in sensitivity to pressure due to differences in subcutaneous tissues and tissue structures. From the perspective of ergonomics, Kohara et al. [23] suggested that the human body’s perception of pressure can be divided into dull parts and sensitive parts. The dull parts can withstand greater pressure, and the sensitive parts can only feel comfortable when the pressure is low. Designing different support conditions for different areas can better ensure user comfort [24,25,26].

Existing research on pillow comfort is still at the initial theory establishment stage and is currently facing several problems. First, it is unclear whether there is comfort demand disparity at different head and neck regions. Second, the head and neck of the human body are typically regarded as a whole, which ignores the difference in pressure sensitivity of contact surfaces in different areas. Third, the use of BP neural network or support vector machine and their derivative algorithms can effectively predict the comfort evaluation of existing pillows to a certain extent, but because the processes of these two algorithms are hidden and cannot be reversed, it is difficult to directly apply the optimal solution of the head and neck support scheme to product development. Fourth, the applicability of the evaluation model is limited, and certain errors will occur when it is extended to different physiques [27].

This paper proposes a head and neck support model with partitioned matching based on the body pressure distribution matrix. The proposed model was divided into two modules: partition body pressure distribution index matching and overall matrix similarity matching. This study was carried out in three stages. First, objective pressure distribution ergonomic experiments and subjective comfort evaluation experiments were performed on existing products. Combining the objective and subjective results, the pressure distribution matrix of the ideal support surface was obtained. Then, the support surface partitions were determined by fuzzy clustering. By combining the body pressure distribution index of each partition and the ideal body pressure distribution matrix, the ideal support model of the head and neck area in the sleeping position was constructed. Second, in order to realize the partition support model, we combined the key ergonomic parameters and material physical quantities of different groups to establish the standard sleep pillow prototype. Third, we performed an experiment on the ergonomic comfort of the standard sleep pillow prototype, observed the sleep comfort with the support of the standard pillow, and verified the model to extract relevant information.

## 2. Experiment of Head and Neck Body Pressure Distribution in Recumbent Position

### 2.1. Head and Neck Body Pressure Distribution in Recumbent Position

Body pressure distribution is one of the most intuitive indicators of pillow support comfort. Reasonable pressure distribution is the key to ensuring comfort [28]. It is also an important basis for the partition of the supporting surface of the sleep pillow. To remove the interference of muscle activation, it is necessary to ensure that the subjects’ muscles are fully relaxed, so the surface EMG (sEMG) was used as the observation value.

#### 2.1.1. Subjects

To ensure the accuracy of the experiment and the generalizability of the results, six male and female graduate students with healthy bodies and normal cervical curvatures were recruited. In order to reduce the interference caused by differences in body shape, the physical fitness levels of the subjects were relatively close. The basic information of the subjects is as follows: for males, age 25 ± 2 years old, height 172.1 ± 4.3 cm, and weight 60.2 ± 4.2 kg; for females, age 25 ± 2 years old, height 161.1 ± 3.1 cm, and weight 51.4 ± 2.8 kg.

Due to its wide elastic adjustable range and good shaping freedom, memory foam is the most common material of current ergonomic pillows [29,30]. After comparing 32 pillows on the market, 7 memory foam ergonomic sleeping pillows with the highest user reviews were selected as samples. After comparing the 32 pillows that sold over 10,000 pieces on Taobao (the largest online sales platform in China) from 30 November 2019 to 30 November 2020, 7 of the memory foam ergonomic sleeping pillows with the highest comfort rating in their user feedback were selected as samples. The shapes and basic parameters of the pillows are shown in Table 1.

#### 2.1.2. Apparatus

In the experiment, a multi-channel physiological signal acquisition system (MP150, BIOPAC Inc., Goleta, CA, USA) was used to collect the EMG signals of targeted muscle activity. Disposable, bipolar Ag/AgCl electrode pads were used, which had a gel-based filling solution, a diameter of 30 mm, and an inter-electrode distance of 2 cm. The frequency of EMG data sampling was set to 1000 Hz.

The experiment used the American Tekscan body pressure measurement system (BPMS) with a 1200-point flexible pressure pad, which has superior stability, accuracy, and measurement density compared to related products.

### 2.2. Experimental Procedure

**Figure 1:**
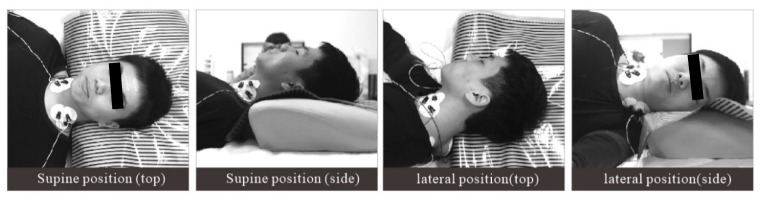
Pressure distribution experiment under the monitoring of EMG signal.

Under the environmental conditions of a room temperature of 24 °C, humidity of 45%, and a noise level of 24 dB, the subjects maintained supine and lateral positions for 5 min each. In the supine position, the subjects were instructed to keep their arms on both sides of the body. In the lateral position, the subjects lay on their right side, and they were allowed to bend their knees. The body pressure distribution data of the head and neck were collected. A period of two minutes of rest was provided during the position switch to reduce fatigue. To eliminate the influence of continuous testing on subjective evaluation, the subjects were allowed to get up and move slightly for 5 min between tests of different pillow types. During the test, to ensure that the subjects’ muscles were fully relaxed, the upper trapezius (UT) and sternocleidomastoid (SCM) muscles, which are most closely related to the head posture, were selected to acquire EMG signals as observation data [31]. The electrode was placed over the UT muscle at 50% of the distance from the acromion to the C7 vertebra, and the bilateral SCM muscle electrode was placed at the one-third point of intersection from the bottom edge of the mastoid to the upper sternal tangent. As shown in Table 2, when the EMG showed electrical resting, the muscle was considered to be fully relaxed [32], and the pressure distribution data were considered to be valid. The experimental method is shown in Figure 1.

After the testing, the subjects were asked to fill out the experience evaluation form to evaluate the comfort of the seven types of pillows in the two sleeping positions. The evaluation indicators included four items: softness, wrapping, support, and fit. Each item was scored on a 5-point scale, with 1 being the least comfortable and 5 being the most comfortable, for a maximum possible score of 20 points.

### 2.3. Data Processing and Analysis

For the construction of the head and neck occipital support model with partitioned matching in the supine and lateral positions, we calculated the body pressure distribution index, determined the ideal pressure distribution matrix, and delineated the partitions.

#### 2.3.1. Body Pressure Distribution Index Calculation

As a physical quantity that characterizes the distribution of body pressure, the body pressure index serves as a bridge between the body pressure test results and the subjective comfort evaluation. The selection of an appropriate body pressure index can result in a better correlation between the objective test results and the subjective comfort evaluation results. This study selected average pressure, peak pressure, maximum pressure gradient, and average pressure gradient as body pressure indicators.

(1) Average Pressure *Pv* (kPa)

The average pressure *Pv* is the arithmetic mean value of all pressure points (test points), namely:

(1) $$Pv=\frac{1}{N}\overset{N}{\underset{i}{\sum }}P_{i}$$

where *N* is the number of test points, and *P_i_* is the pressure of each test point. The average pressure is the tendency of the pressure value to concentrate, which to some extent reflects the overall effect of the pillow on the head and neck support.

(2) Peak pressure *P_m_* (kPa)

The peak pressure *P_m_* is the maximum value of the pressure at all measuring points, which is denoted as:

(2) $$P_{m}=max\left(P_{1},P_{2},\ldots ,P_{n}\right)$$

where *n* is the number of measuring points, and *P_i_* is the pressure of each measuring point.

(3) Pressure gradient *G* (kPa/cm^2^)

Pressure gradient is the rate of change of pressure along a certain direction. It reflects the degree of difference between adjacent pressures. The greater the pressure gradient, the greater the sensitivity of the human body to pressure stimulation. Both the maximum pressure gradient and the average pressure gradient were considered.

Maximum pressure gradient *G_m_* (kPa/cm^2^):

(3) $$G_{i}=\frac{\sum_{j=1}^{N_{p}}\left[p\left(x_{i},y_{j}\right)-p\left(x_{i-1},y_{j}\right)\right]}{l}$$

(4) $$G_{m}=max\left(G_{1},G_{2},\ldots ,G_{n}\right)$$

where *G_i_* is the pressure gradient value of the *i*-th measuring point, *p*(*x_i_*, *y_j_*) is the pressure of the *j*-th measuring point in the *i*-th row, *N_p_* is the number of pressure points, *l* is the distance between two measuring points, and *n* is the number of measuring points.

Average pressure gradient *G_v_* (kPa/cm^2^):

(5) $$G_{v}=\frac{1}{N}\sum_{j=1}^{N}(G_{i}).$$

The average pressure gradient is the arithmetic mean of the pressure gradient of each pressure point.

#### 2.3.2. Determination of Ideal Pressure Distribution Matrix and Delineation of Partitions

In the experimental results, the pressure distribution matrix with higher comfort evaluation was relatively more in line with the subjects’ corresponding head and neck support needs. To a certain extent, it characterized the approximate ideal pressure distribution matrix. To reduce the influence of the individual score differences of the subjects, the pressure distribution maps were sorted according to the subjective comfort evaluation, and the average value of the pressure distribution matrices of the better evaluation was calculated. The new pressure distribution matrix can be regarded as a relatively universal approximate ideal pressure distribution matrix.

The similarity with the ideal pressure distribution matrix is an important basis for evaluating whether the object restores the ideal bearing surface. Matrix similarity was calculated using the cosine similarity equation:

(6) $$imilarity=\frac{A*B}{\left\|A\|\|B\right\|}=\frac{\sum_{i=1}^{n}A_{i}*B_{i}}{\sqrt{\sum_{i=1}^{n}{\left(A_{i}\right)}^{2}}*\sqrt{\sum_{i=1}^{n}{\left(B_{i}\right)}^{2}}},$$

where *A_i_* and *B_i_* represent the value of each pressure point of the ideal pressure distribution matrix and the actual pressure distribution matrix of the prototype, respectively. A calculated value closer to 1 indicates that the two matrices are closer.

Partition body pressure distribution index matching is a very important part of the pillow support model. How to determine the partition is particularly critical. The change of pressure values of each point in the ideal pressure distribution matrix are continuous and smooth, with no obvious boundaries on the image. Therefore, this study used the fuzzy clustering algorithm to cluster the pressure values of each point in the matrix, in order to clearly show the boundaries of areas, and take them as the basis for dividing the pillow surface.

FCM was performed on the approximate ideal pressure distribution matrix. According to the obtained results, the pressure distribution map can be partitioned intuitively and effectively. The mathematical model is as follows:

(7) $$J_{m}\left(\mu ,V\right)=\sum_{i=1}^{n}\sum_{k=1}^{c}\mu_{ik}^{m}x\|x_{i}-v_{k}{\|}^{2},$$

where *n* represents the number of pressure points, and *c* represents the number of partitions. To make the conclusion clear, the value of *c* was set to 3 in this study. Additionally, *v_k_* represents the cluster center of the *k*-th category; *μi_k_* represents the member degree of the *i*-th sample belonging to the *k*-th category; ‖*x_i_*−*v_k_*‖^2^ represents the squared Euclidean distance from the sample *x_i_* to the cluster center *v_k_*; and *m* represents the fuzzy index, which is generally set as 2.

#### 2.3.3. Partition Pressure Sensitivity Weight

According to the physiological anatomy of the human head and face, the subcutaneous tissue includes bones, muscles, nerves, and blood vessels. There are significant differences in the pressure sensitivity of various human tissues [33]. As shown in the partition support model, areas with higher sensitivity have more stringent requirements for the restoration of the ideal pressure value. In addition, the pressure sensitivity weight also represents the user’s degree of attention to this partition when using pillows. Therefore, it is necessary to judge the pressure sensitivity weight of each partition of the pillow surface.

The fuzzy analytic hierarchy process (FAHP) in operations research was used to construct the judgment matrix of different levels of factors to calculate the weight of each partition item. Experts in related fields were invited to compare the compression sensitivity of the partitions in pairs and use a nine-level scale to score according to the strength, where the highest sensitivity is 9 and the lowest is 1. We used *v* to indicate that there were *n* partitions having the weighted judgment matrix:

(8) $$M^{C}\left[\begin{array}{ccccc} v11 & v12 & v13 & \ldots & v1n\\ v21 & v22 & v23 & \ldots & v2n\\ \ldots & \ldots & \ldots & \ldots & \ldots \\ vn1 & vn2 & vn3 & \ldots & vnn \end{array}\right]$$

where *V_ij_* is the comparison result of the pressure resistance of the partition *i* and the partition *j*.

The matrix solution is as follows:

(9) $${W^{C}}_{i}=\frac{\sum_{j=1}^{n}vij+n/2-1}{n(n-1)},(1\leq i\leq n),\;$$

where *W^C^_i_* is the weight of the *i*-th partition of the C layer, *n* is the number of partitions, and *V_ij_* is the strength of the pressure resistance of the partition *i* and the partition *j*.

Using the above formula to calculate the weights of the middle layer and the bottom layer as {*W^B^*} and {*W^C^*}, the final weight value W* is:

(10) $$W^{*}=\{{W^{B}}_{1}\;\times \;{W^{C}}_{1},\;{W^{B}}_{2}\;\times \;{W^{C}}_{2},\ldots ,\;{W^{B}}_{i}\;\times \;{W^{C}}_{i},\ldots ,\;{W^{B}}_{n}\;\times \;{W^{C}}_{n}\},\;(1\leq i\leq n).$$

If each matrix passes the consistency test, then W* is the final weight for the pressure tolerance of each partition.

#### 2.3.4. Sleeping Comfort Calculation

After completing the above calculations, considering the actual situation, the comprehensive comfort calculation formula is as follows:

(11) $$Comfort=\sum_{i=1}^{n}\left[x_{i}*w_{i}\right],$$

where *x_i_* represents the comfort evaluation of the *i*-th partition, and *w_i_* represents the weight of the comfort of the *i*-th partition in the total evaluation result.

The comprehensive comfort index can be used to obtain the comprehensive comfort score more accurately on the basis of the evaluation status of the partitions.

## 3. Multi-Partition Support Model Construction and Prototype Production

### 3.1. Sleep Pillow Partition Based on Body Pressure Distribution

#### 3.1.1. Ideal Sleep Head and Neck Pressure Distribution

**Figure 2:**
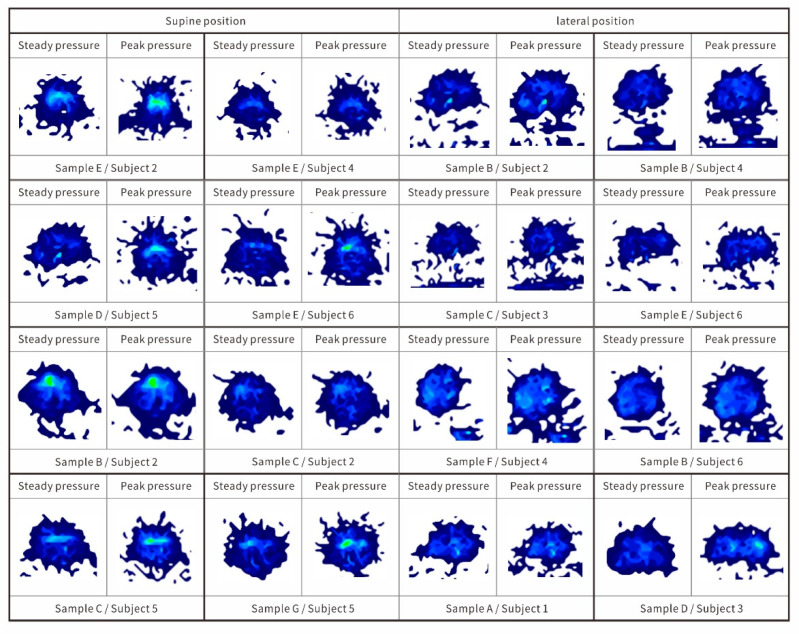
The highest comfort evaluation of the eight groups of supine and lateral pressure distribution map.

Each subject tried all 7 kinds of pillows, so there were 42 sets of results. The body pressure distribution of the top 10% of the four samples in the comparative test is shown in Figure 2, and the key body pressure distribution indicators are shown in Table 3. The body pressure distribution of the top eight samples in the comparative test is shown in Figure 2.

After obtaining the statistics of comfort scores, it can be found that only the top four results scored higher than 4 in all items (more comfortable or above). The key body pressure distribution indicators are shown in Table 3.

The pillow types and subjects with the highest subjective evaluation of comfort in the lateral position were A/S2, B/S4, G/S3, and E/S5. The average pressure between samples ranged from 1.62 to1.95 kPa, the maximum pressure ranged from 7.32 to 8.65 kPa, the maximum pressure gradient ranged from 1.93 to 2.36 kPa/cm^2^, and the average pressure gradient ranged from 0.23 to 0.27 kPa/cm^2^. The pillow types and subjects with the highest subjective evaluation of comfort in the supine position were E/S2, E/S4, D/S5, and E/S6. The average pressure between the samples ranged from 1.72 to 2.27 kPa, the maximum pressure ranged from 7.52 to 9.21 kPa, the maximum pressure gradient ranged from 1.95 to 3.01 kPa/cm^2^, and the average pressure gradient ranged from 0.22 to 0.38 kPa/cm^2^. The average pressure gradient of the above samples was in the middle of the overall ranking and showed a certain degree of clustering. The conclusion here supports the findings of Lee [34] and Chen [35]; namely, a pressure distribution that is too concentrated or too uniform reduces the comfort. It is reasonable to control the average pressure gradient between 0.2 and 0.4 kPa/cm^2^. From the pressure distribution graph, the peak pressure of the pressure distribution graph with the highest subjective comprehensive evaluation was relatively uniform, corresponding to the occipital apex when lying supine and the temporal bone area when lying laterally. It is expected that these two support functions are similar to the position of the seat bone tubercle when supporting the head and neck in the sleeping position [36,37].

Among the sample pillow types, the comfort of the E-type pillow with neck extension support was significantly better than the other pillow types. In addition, most of the subjects reported that the pressure on the auricle had a greater impact on comfort. The experimental results showed that the B, C, and E pillow-type subjective comfort scores of the auricle were relatively good when lying laterally.

#### 3.1.2. Partition and Weight Calculation of Ideal Head and Neck Pressure Distribution

**Figure 3:**
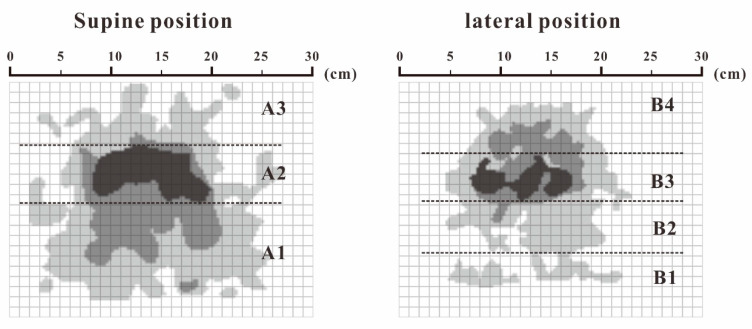
Ideal pressure distribution matrix partition.

By averaging the four pressure distribution matrices with the highest comfort evaluation, an approximate ideal pressure distribution matrix can be obtained. The result of FCM clustering on this matrix is shown in Figure 3. In the supine position, the head and neck support can be divided into three partitions: the posterior neck area (A1), occipital area (A2), and posterior parietal area (A3). In the lateral position, the support can be divided into four partitions: the cervical area (B1), jaw area (B2), temporal bone area (B3), and lateral parietal area (B4), which are shown in Figure 3.

**Figure 4:**
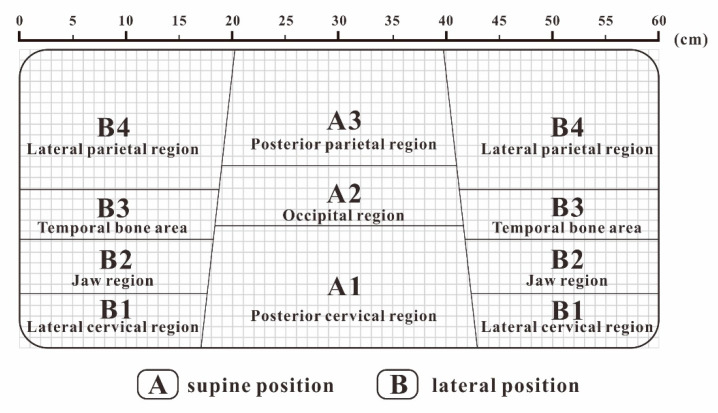
Pillow partition.

Here, we borrowed the concept from Kilincsoy’s method of dividing the body pressure map by the length of the thigh and the length of the spine of the human body and mapping it to the surface of the support surface [38]. We combined the actual conditions of the recumbent ergonomics to map the ideal pressure distribution by partitioning the size of each area on the pillow surface, as shown in Figure 4. We extracted and calculated the above ideal pressure distribution of the body pressure distribution index of each partition, as shown in Table 4.

**Figure 5:**
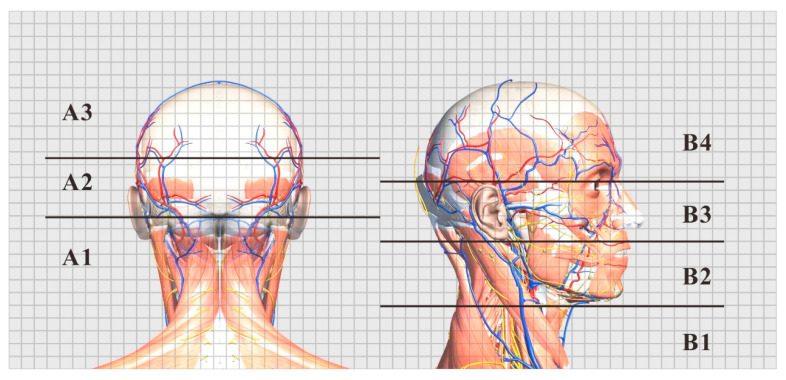
Subcutaneous tissue and structure of each partition.

**Figure 6:**
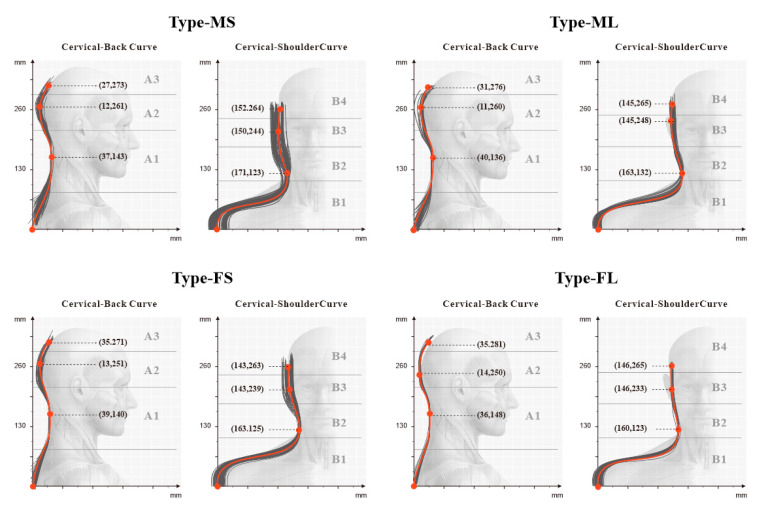
The median coordinates of the ergonomic nodes of the head and neck contours of the four groups of people.

We used the medical anatomy software (Complete Anatomy) to display the subcutaneous tissue of the head and face and divided the area according to the ideal support zone size, as shown in Figure 5. In accordance with the method described in Section 2.3.3, we invited 10 experts in related fields, including 4 chief physicians, 4 head analysis researchers, and 2 senior pillow designers, to compare and evaluate the pressure sensitivity of 7 partitions in the 2 sleeping positions with Figure 6 as the object, and the hierarchical analysis was carried out accordingly. The final weight average is shown in Table 5.

As shown from the above table, the temporal bone partition (B3) had the highest weight of 0.213, so the pressure data restoration should be precise for this partition. The posterior parietal area (A3) had a low sensitivity with a weight of 0.082, so the requirements for data restoration of this partition could be relaxed.

### 3.2. Prototype Production of Ideal Support Model

To verify the effectiveness of the ideal head and neck support model more realistically, this study used finite element simulation to verify the model and also made a prototype based on the ideal partition support model to carry out empirical research. The key to the design of an ideal support prototype is to restore the spatial dimensions and equivalent elastic coefficients of the subdivisions of the head and neck support surfaces of different groups of people.

#### 3.2.1. Key Ergonomic Node Coordinates

To verify the universal applicability of the occipital support model with partitioned matching, this study collected 573 ergonomic data samples of the head and neck of Chinese people through the use of a profile ruler and a Martin measurement instrument. To match the natural posture of the human body during sleep, the upright posture was used for the collection of the back profile data, and the shoulder-wrapped posture was used for the collection of edge contour data [39]. We selected both men and women with a height and BMI (kg/m^2^) either less than the 40th percentile or greater than the 60th percentile. A total of 231 samples were categorized into 4 groups: male with small physique (MS), male with large physique (ML), female with small physique (FS), and female with large physique (FL). We obtained the front and side projection contour curves of the head and neck of the four groups of people through data entry. We marked the key ergonomic nodes of each partition in the curve (the comparison between the partitions and the corresponding key ergonomic nodes is shown in Table 6) and calculated the median value of the coordinates of each node in the group, as shown in Figure 6.

#### 3.2.2. Prototype Fabrication of Ideal Support Pillow

**Figure 7:**
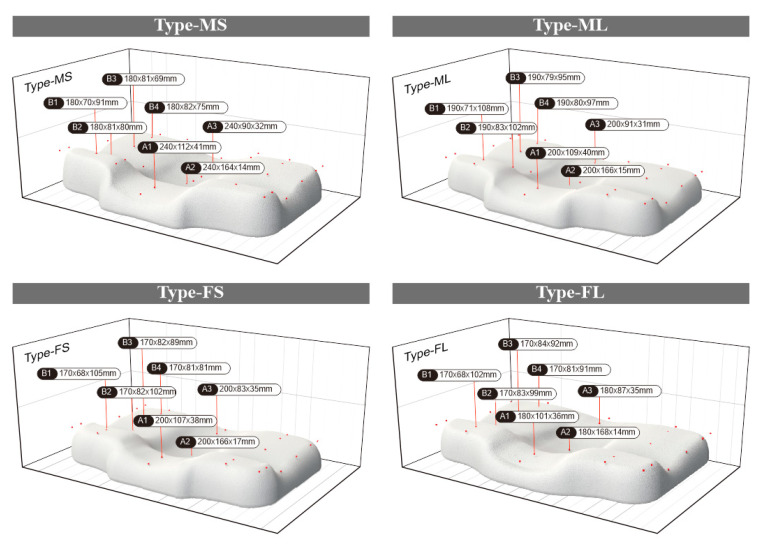
The morphology of zone 11 after four types of pillows are compressed.

By mapping the ergonomic node positions in the average ergonomic contour lines of the above four types of people to the pillow partition model, the shape and size of the pillow after compression was obtained, as shown in Figure 7.

We combined the height of each partition of the pillow after compression with the pressure distribution value of each partition in the standard pressure distribution matrix, and the equivalent elastic coefficient required by each partition was obtained, calculated as follows:

(12) $$k_{i}=\Delta F_{i}/\Delta H_{i},$$

where *k_i_* is the comprehensive stiffness coefficient of the material in the *i*-th partition, Δ*F_i_* is the total pressure of the *i*-th partition, and Δ*H_i_* is the amount of height change.

**Figure 8:**
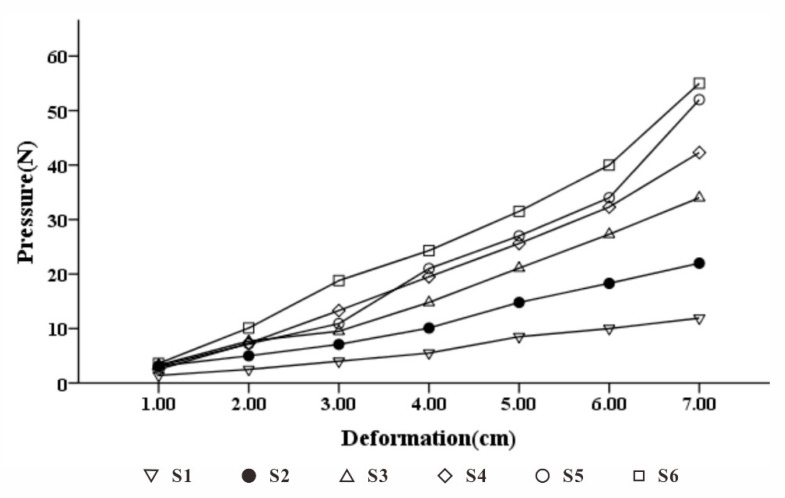
60 D memory foam deformed under pressure with different apertures.

Due to the complex physical properties of foamed materials, the calculation of elastic coefficient is complicated [40]. Here, taking into account the actual production process, this paper made the pore-array structure with different apertures on the material to achieve different elastic coefficient changes under the same volume of material. We chose a commonly used pillow with a thickness of 110 mm [41], a density of 60 D, and a memory foam with a staggered hole pitch of 30 mm. The pore diameter was taken as a variable, and a 5-cm diameter disc end pressure rod was used to simulate head pressure [42]. The relation between the pore diameter and the pressure deformation measured by the experiment is shown in Figure 8. Through linear fitting, it can be seen that within a certain pressure range, ∆*F_i_* and ∆*H_i_* basically presented a linear relationship. Therefore, the equivalent elasticity of the memory foam material after perforation was constant. The coefficient *k_i_* is shown in Table 7.

**Figure 9:**
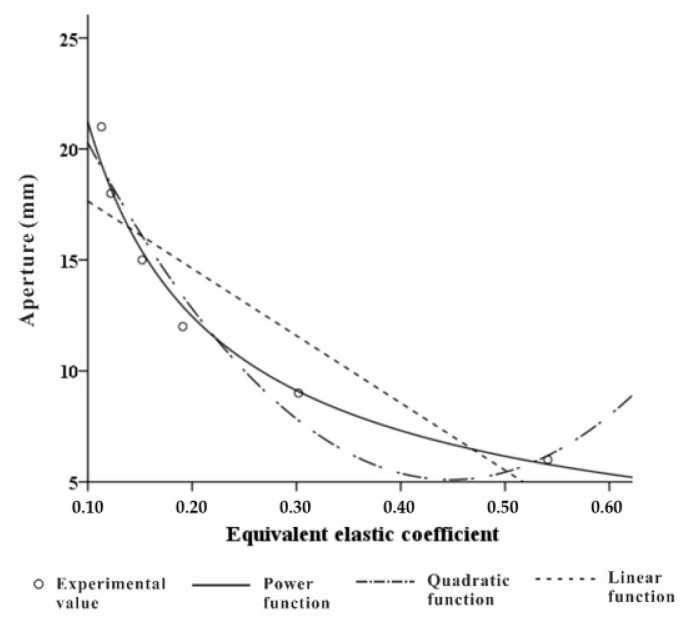
Pore diameter-equivalent elastic coefficient regression curve.

Through the actual measurement, the aperture was used as the independent variable, and the equivalent elastic coefficient *k* value was linearly regressed, as shown in Figure 9. The power function, expressed as

(13) $$d=3.613k^{-0.769}\;(6\;\leq \;d\;\leq \;21),$$

had a higher degree of fit.

**Figure 10:**
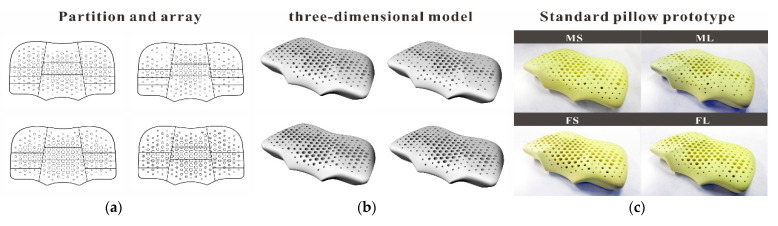
(**a**) Hole array distribution. (**b**) 3D model. (**c**) Prototype.

In principle, by combining the relevant data in the ideal pressure distribution matrix and the pillow shape after compression, the above regression equation can be used to calculate the pore-array distribution that meets the pressure distribution requirements in any pillow shape, with a height of 110 mm and a density of 60 D. For example, we substituted the elastic coefficient *k_i_* required for each partition and used the RHINO parameterized modeling tool to obtain the mapping pore-array distribution, as shown in Figure 10a,b. According to this, 60 D polyester fiber is used as the material to make the standard ideal. The support pillow prototype is shown in Figure 10c. We detected the equivalent elastic coefficient value of the key ergonomic node mapping position in each partition, as shown in Table 8.

It can be seen that the *k* value of each partition of the ideal support pillow prototype was not much different from the calculated value, which was in line with our expectations.

## 4. Research on Ergonomics of Multi-Partition ideal Support Pillow

According to the information of the divided population before the prototype was produced, this experiment recruited five healthy individuals with normal cervical spine that fit the partitions as the subjects, as shown in Table 9. We used the corresponding zoning ideal support model sample pillow and carried out a verification experiment with reference to the method described in Section 2 of this article. At the same time, the E-type pillow with the highest comprehensive score in the first experiment was selected as the control pillow. After the test, the subjects were asked to evaluate the overall comfort of each partition.

**Figure 11:**
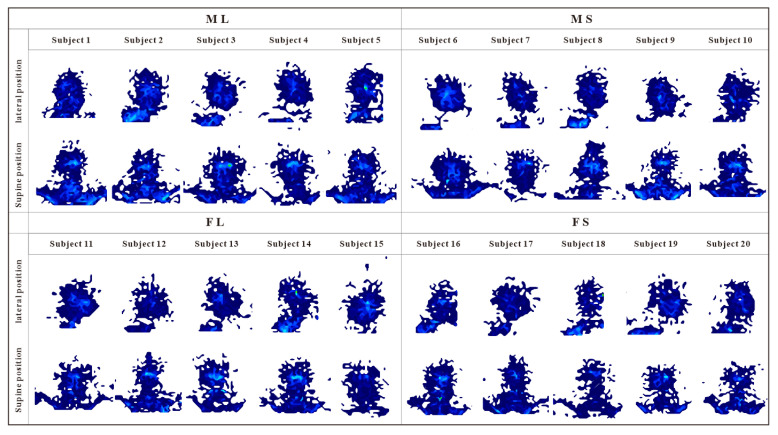
Pressure distribution diagram of prototype.

From the obtained pressure distribution image (Figure 11), the pressure distribution showed good consistency across subjects.

**Figure 12:**
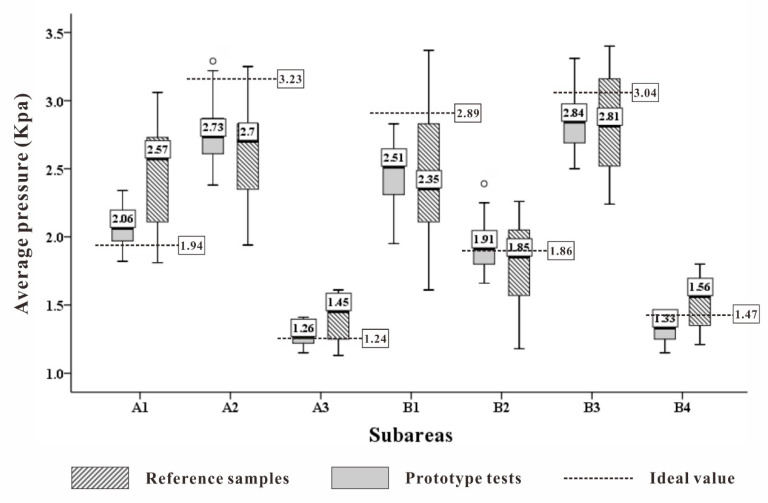

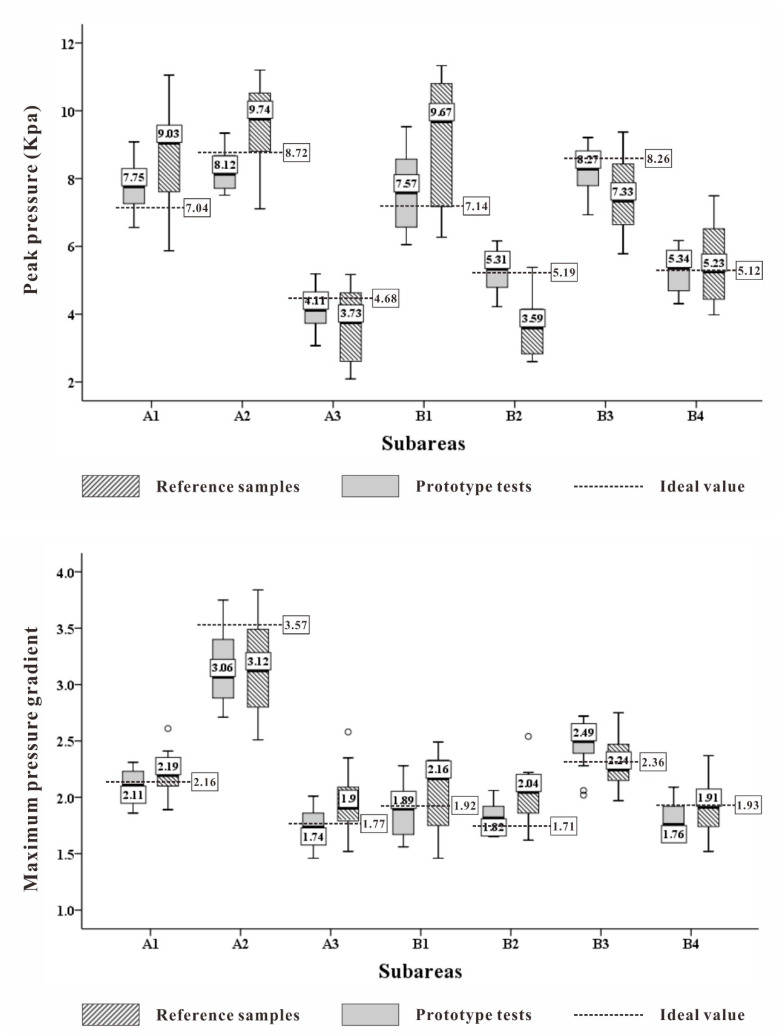

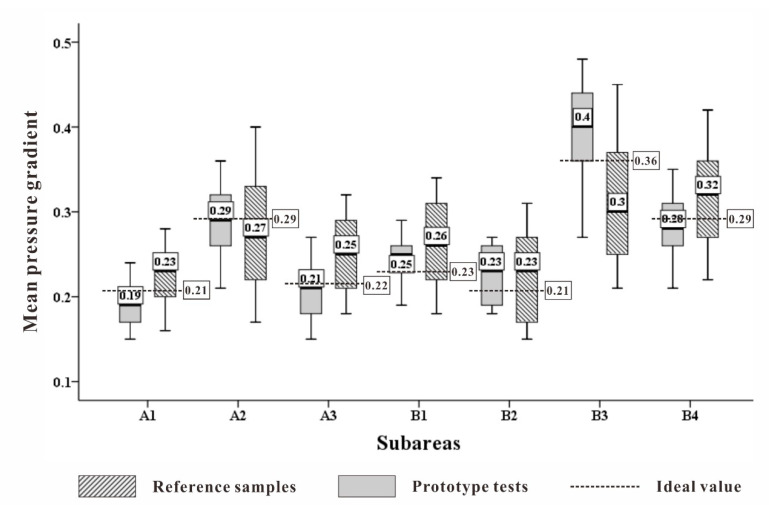
Comparison of body pressure distribution indicators in each partition of the four groups.

By comparing the average pressure, peak pressure, maximum pressure gradient, and average pressure gradient of the prototype and control pillow subjects (Figure 12), the following information can be found. Considering the average pressure, the distribution of values in each area of the prototype is more concentrated, and the median is closer to the ideal value described in Table 4 than that of the control group. In the A3 region, the value is very close, only +0.02. For the peak pressure, the results are similar to the average pressure, the median values of each area of the prototype are closer to the ideal value, especially in the B2 and B3 areas, and the difference is +0.12 and +0.05. In the maximum pressure gradient, the advantages of the prototype in A3, B1, and B2 areas are −0.03, −0.03, and +0.05, respectively. On the contrary, the values in the B4 area are slightly worse than those in the control group. On the average pressure gradient, the distribution and median value of each zone of the prototype are better than those of the control group, but the difference is not big, and the A2 zone is the closest, being almost equal to the ideal value. In summary, the data distribution of the prototype was more convergent and concentrated. The median value was close to the ideal value, especially in the B2 and B3 areas with higher sensitivity weights, which indicated excellent approximation capabilities. The partition prototype was better than the control pillow in reducing the ideal body pressure index in each partition. Especially in the two indicators of average pressure and peak pressure, the performance improvement was more obvious. In the two data sets of maximum pressure gradient and average pressure gradient, the data distribution was relatively concentrated, and the distribution gap was small. The sensitivity of these two indicators was relatively poor when deciding whether to restore the ideal body pressure matrix. In the actual development of pillow, the average pressure and peak pressure should be given priority.

In addition to restoring the ideal body pressure indicators in each partition, the similarity of the pressure distribution matrix is also an important basis for evaluating whether the prototype has restored the ideal support surface. The matrix similarity is calculated by Equation (6).

**Figure 13:**
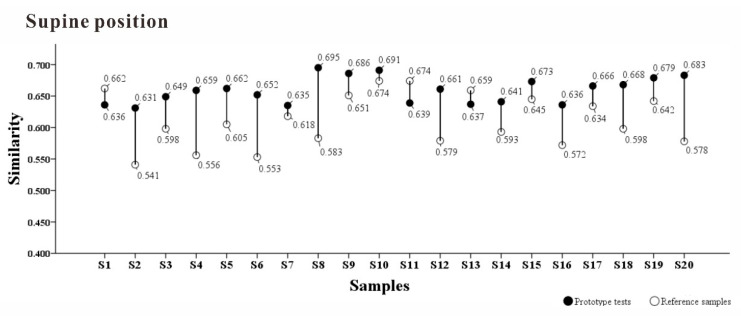

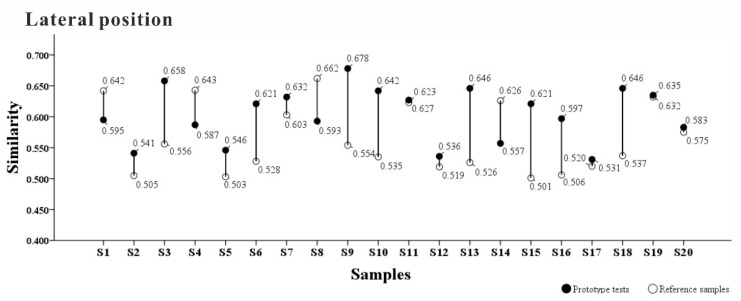
The similarity between the actual measured sample and the reference sample of the prototype and the ideal pressure distribution.

The results are shown in Figure 13.

Compared with the control pillow, the partition support model reproduced the ideal pressure distribution matrix more accurately in different postures. In the supine position, the similarity of the pressure distribution matrix with the ideal pressure distribution matrix was relatively uniform across all subjects, indicating that the ideal support for the supine position was achieved. In comparison, the lateral position showed large fluctuations, and the similarity of the prototype pillow was not different from that of the control pillow for some individuals (4/20) and was even slightly lower than that of the control pillow for some other individuals (4/20). Compared with the supine position, the support surface requirements for the lateral position were more complicated and required more investigation. In the case of lateral position, four subareas may not be enough. It may need to be further subdivided.

**Figure 14:**
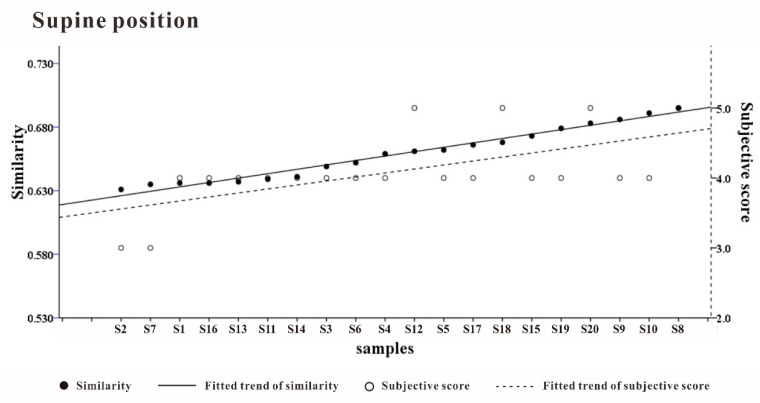

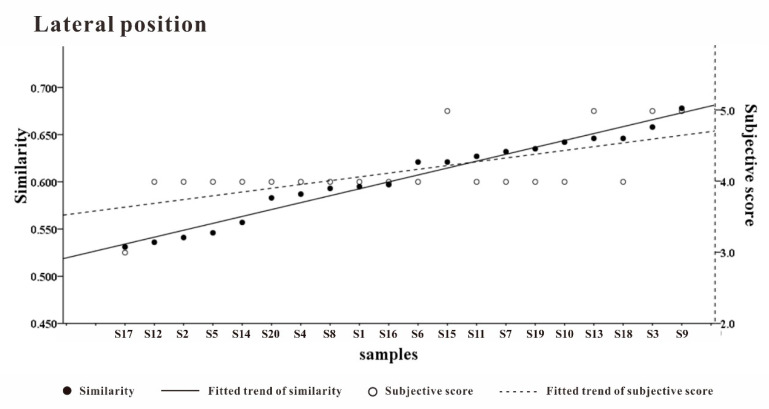
Ideal pressure distribution similarity ranking and subjective evaluation trend.

We also sorted the prototypes according to the similarity with the ideal pressure distribution matrix from low to high and compared the subjective comfort scores, as shown in Figure 14. The similarity trend and the comfort evaluation showed a high degree of consistency. The similarity with the ideal pressure distribution matrix characterized the comfort evaluation to a considerable extent. Whether the ideal pressure distribution matrix can be reproduced is an important indicator for evaluating the comfort of pillows. In the development stage, the comfort can be predicted by calculating the similarity between the target and the ideal pressure distribution matrix by finite element analysis.

**Figure 15:**
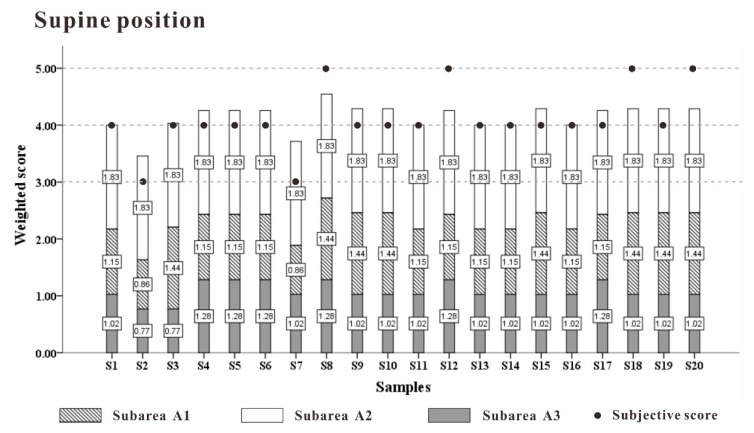

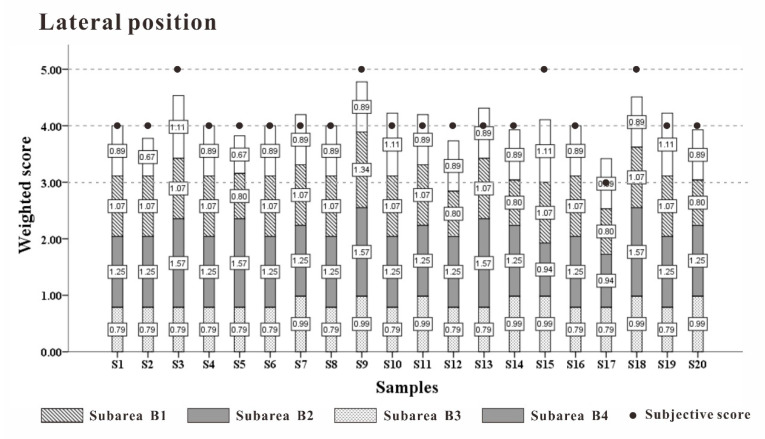
Comparison of weighted comprehensive evaluation of each recumbent position.

According to Equation (11), the comprehensive weighted comfort evaluation in each recumbent position was calculated. The comparative subjective score is shown in Figure 15. The two values showed a high degree of consistency. The comfort weight value of each partition was highly reliable.

## 5. Conclusions

The main conclusions of this research are as follows:

(1) Through the body pressure distribution experiment, the average pressure distribution matrix of several samples with the highest comfort score can be obtained, and the approximate ideal pressure distribution matrix can be obtained. The similarity with the ideal body pressure distribution matrix can effectively characterize the comfort evaluation to a certain extent.

(2) The ideal pressure distribution matrix can be divided by the fuzzy clustering algorithm into three partitions for the supine position: posterior neck area, occipital area, and posterior parietal area, and four partitions for the lateral position: cervical area, jaw area, temporal bone area, and lateral parietal area. The ideal body pressure distribution index of each partition is shown in Table 4.

(3) The analytic hierarchy process based on expert evaluation of head and facial tissues can determine the pressure sensitivity weight of each partition. It expresses the accuracy requirements of the partition to restore the ideal pressure distribution, and it is also the weight used to calculate the overall comfort evaluation. Among them, the highest weight of the temporal bone area is 0.213, which requires special attention, the lowest weight of the posterior parietal bone area is 0.086, and the standard can be appropriately relaxed.

(4) We constructed an ideal head and neck support model based on knowledge of ideal body pressure distribution matrix, partition body pressure distribution indicators, and pressure sensitivity weights. Combining the support model with the regression function of the key node coordinates of the population partitions and the equivalent elastic coefficient of the material, a prototype can be produced to effectively reproduce the ideal pressure distribution matrix and the partition body pressure distribution index in different populations. This is of great significance to the design and development of pillows.

## 6. Future Prospects

The ideal pressure distribution matrix described in this study was derived from the existing pillow-shaped body pressure distribution experiment, which has several major limitations, and it is possible that a better support plan could be developed. Therefore, the obtained ideal pressure distribution matrix can only be considered to be relatively good to a certain extent. In addition, the classification of the population in the prototype verification stage is relatively rough, as it only considers the size of the ergonomics and fails to refine the impact of individual differences in dimensions such as age, head shape, and chronic diseases. In future studies, we plan to further improve the ideal support model of the head and neck area so that it has the capability to iteratively approximate the ideal pressure distribution matrix and global optimization, and so that the pincushion output can be accurately positioned to various subdivided populations.

## Figures and Tables

**Table 1:** Sample parameters of seven types of ergonomic sleep pillows.

	A	B	C	D	E	F	G
	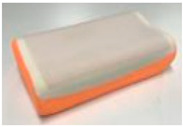	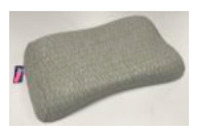	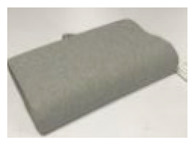	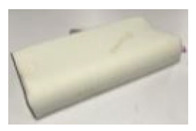	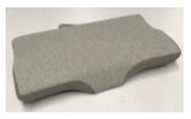	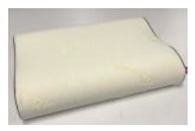	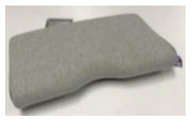
Length (mm)	500	600	500	600	600	600	500
Width (mm)	350	350	300	300	350	400	300
Height (mm)	120/120	90/110	70/100	70/100	60/110	100/120	60/110
Density	60D	40D	60D	60D	40D	40D	60D
Material	polyurethane	polyurethane	polyurethane	polyurethane	polyurethane	polyurethane	polyurethane

**Table 2:** Instructions for the location of the muscle to be tested and the electrode sheet to be pasted.

Name of the Target Muscle	Trapezius (UT)	Sternocleidomastoid (SCM)
Electrode location	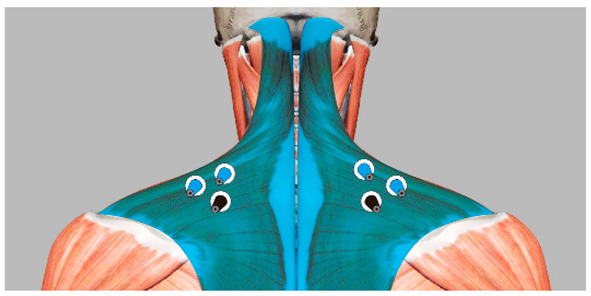	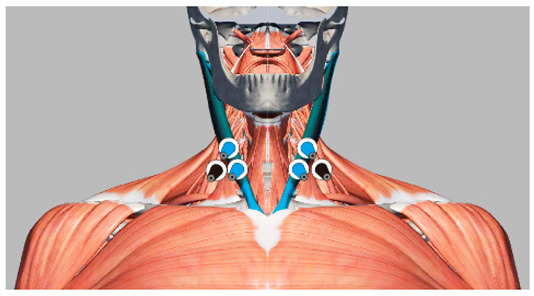

**Table 3:** Body pressure distribution index of top four results.

	Lateral Position				Supine Position			
	B/S2	B/S4	C/S3	E/S6	E/S2	E/S4	D/S5	E/S6
Comfort score	17	16	16	16	17	17	16	16
Average pressure	1.71	1.82	1.95	1.62	2.27	1.72	2.15	2.18
Peak pressure	7.72	8.65	7.52	7.36	7.52	7.98	8.53	9.21
Maximum pressure gradient	2.05	1.93	2.36	1.96	1.95	2.14	2.18	3.01
Mean pressure gradient	0.27	0.23	0.25	0.27	0.31	0.22	0.22	0.38

**Table 4:** Partition ideal body pressure distribution index.

	A1	A2	A3	B1	B2	B3	B4
Average pressure	1.94	3.23	1.24	2.89	1.86	3.04	1.47
Peak pressure	7.04	8.72	4.68	7.14	5.19	8.26	5.12
Maximum pressure gradient	2.16	3.57	1.77	1.92	1.71	2.36	1.93
Mean pressure gradient	0.21	0.29	0.22	0.23	0.21	0.36	0.29

**Table 5:** Weight of compressive coefficients in each partition.

	Supine Position			Lateral Position			
	A1	A2	A3	B1	B2	B3	B4
Average weight	0.146	0.092	0.082	0.151	0.182	0.213	0.134

**Table 6:** The partition node corresponding to each partition.

A1	A2	A3	B1	B2	B3	B4
Posterior Cervical Region	Occipital Region	Posterior Parietal Region	Lateral Cervical Region	Jaw Region	Temporal Bone Region	Lateral Parietal Region
The fourth cervical vertebra	Occipital eminence	Parietal foramen	Intersection of larynx and neck edge	Angle of mandible	Zygomatic arch	Parietal tuber

**Table 7:** Measured equivalent elastic coefficient under different pore diameters (*d*).

	S1	S2	S3	S4	S5	S6
*d* (mm)	6	9	12	15	18	21
*k*	0.541	0.302	0.191	0.152	0.122	0.113

**Table 8:** Error of equivalent elastic coefficient of each partition for men with small physique.

	A1	A2	A3	B1	B2	B3	B4
*k* (Calculated)	0.13	0.11	0.15	0.33	0.26	0.12	0.14
*k* (actual)	0.14	0.10	0.16	0.34	0.24	0.11	0.15
Error	7%	9%	7%	3%	11%	8%	7%

**Table 9:** Details of experiment subjects.

	Gender	Age	Height (cm)	Weight (kg)
MS	male	age 25 ± 5	168.4 ± 9.6	57.3 ± 8.1
ML	male	age 25 ± 5	182.1 ± 7.3	72.3 ± 7.1
FS	female	age 25 ± 5	153.5 ± 6.8	48.4 ± 6.7
FL	female	age 25 ± 5	173.1 ± 5.2	64.7 ± 5.4

## Data Availability

The data presented in this study are available on request from the corresponding author. The data are not publicly available due to restrictions eg privacy and ethical.
